# A novel solid-state approach for enhancing the antimicrobial and colorimetric properties of pine sawdust using selenium nanoparticles

**DOI:** 10.1038/s41598-026-42703-9

**Published:** 2026-03-27

**Authors:** Nancy Zaghloul, Mohamed Abd El-Twab, Khaled Sayed-Ahmed

**Affiliations:** 1 Faculty of Applied Arts, Department of Interior Design and Furniture, Damietta University, Damietta, 34512 Egypt; 2 Department of Agricultural Biotechnology, The Center for Excellence in Research of Advanced Agricultural Sciences, Faculty of Agriculture, Damietta University, New Damietta, 34517 Egypt; 3 Department of Chemistry, Faculty of Science, New Mansoura University, New Mansoura, 35522 Egypt

**Keywords:** Sawdust modification, Selenium nanoparticles, Antimicrobial activity, Color enhancement, Solid-state synthesis, Biotechnology, Environmental sciences, Materials science, Microbiology, Nanoscience and technology

## Abstract

A novel and sustainable approach was developed to fabricate multifunctional pine sawdust (PS) with enhanced antimicrobial and coloration properties through the in-situ synthesis and deposition of selenium nanoparticles (SeNPs) on the sawdust surface for the first time. Sodium hydrogen selenite and ascorbic acid were employed as precursor and reductant, respectively, using a solid-state reduction technique at room temperature. The solid-state reduction method enables a one-step, solvent-free synthesis of SeNPs directly on sawdust, in line with green chemistry principles. Unlike liquid-phase methods, the solid matrix limits nucleation and growth, minimizing NPs mobility and therefore aggregation and agglomeration without the need for stabilizers. Transmission electron microscopy (TEM) revealed that the SeNPs were uniformly distributed on PS surfaces in spherical form, with sizes ranging from 3 to 68 nm depending on precursor concentration. X-ray diffraction (XRD) confirmed the presence of crystalline SeNPs and successful deposition on the PS. Colorimetric analysis demonstrated that SeNPs imparted vivid orange shades to PS, with color strength (K/S) increasing proportionally with SeNP concentration up to an optimal level, beyond which aggregation decreased color intensity. The PS deposited with SeNPs (SeNPs-PS) exhibited remarkable antimicrobial activity against both Gram-positive (*Bacillus cereus*,* Micrococcus albus*) and Gram-negative (*Escherichia coli*,* Pseudomonas aeruginosa*) bacteria, as well as potent antifungal effects against *Aspergillus niger*, *Aspergillus flavus*, and *Aspergillus ochraceus*. The strongest inhibition zones (up to 38 mm) were observed for *A. niger*. Overall, this eco-friendly fabrication route transforms waste sawdust into a multifunctional bio-composite with promising antimicrobial and aesthetic applications in sustainable materials and wood-based industries.

## Introduction

The construction industry faces critical challenges due to carbon emissions, rising costs, and the potential scarcity of raw building materials, posing threats to global environmental sustainability and construction practices. To address these issues, many countries are adopting modern strategies to promote greener construction through the use of recycled and eco-friendly materials, including waste products, reclaimed wood, and sawdust^1,2^.

Sawdust, a common by-product of furniture production, is abundant and holds significant potential for sustainable reuse. Despite its frequent underutilization, innovative applications of sawdust-based materials are gaining recognition for reducing waste and mitigating environmental harm^3,4^. Sawdust is recyclable in the production of lightweight construction materials and renewable energy resources, highlighting its value as a sustainable material^5^. These advancements contribute to global sustainability goals by transforming wood waste into economically and environmentally beneficial products through waste reduction, resource conservation, and improved energy efficiency^6^.

One noteworthy innovation is the production of wood-based materials like particleboards made from sawdust. These boards present a sustainable alternative to traditional construction and furniture materials by reducing landfill waste and dependence on fresh wood resources^3^. Lightweight, cost-effective, and mechanically robust, these boards are suitable for load-bearing applications while offering thermal and acoustic insulation benefits, enhancing their functional value in interior construction^5,6^. Thus, sawdust particleboards combine sustainability, functionality, and economic viability, making them an appealing option for modern construction and furniture applications.

However, sawdust particleboards face limitations, including susceptibility to microbial colonization due to their organic composition and porous structure. This vulnerability often leads to aesthetic degradation and weakened structural integrity, especially in moist environments^7,8^. Additionally, nutrient-rich extractives in wood encourage microbial growth, limiting their use in settings requiring stringent hygiene standards^9^. Most sawdust-based particleboards are manufactured using urea-formaldehyde (UF) adhesives, which, while cost-effective and commercially available, suffer from limited durability and moisture resistance, restricting their applications^10,11^. Furthermore, phenol-based adhesives emit formaldehyde and other volatile organic compounds (VOCs), raising significant environmental and health concerns^11,12^.

Advanced treatments, such as bioprotective agents and nanoparticle (NP)-infused varnishes, have shown potential to address these challenges by reducing microbial susceptibility and enhancing the durability of wood-based products^13,14^. Nanotechnology has significantly improved particleboard properties by integrating NPs into resins and boards. For instance, nanoclay improves durability and fire resistance without compromising mechanical properties when added to urea-formaldehyde adhesives^15^. Zinc oxide NPs enhance dimensional stability and moisture resistance in wood composites^16^. Similarly, nano-alumina and nano-silica have been shown to improve dimensional stability and reduce formaldehyde emissions^17^.

Green-synthesized NPs have emerged as eco-friendly solutions for enhancing material properties. These biologically derived NPs, created from natural sources, reduce environmental and health risks while improving the mechanical properties and durability of materials^18^. Green NPs also enhance resistance to moisture and microbial decay, addressing limitations of traditional wood panels^19^. Their use aligns with circular economy principles, offering innovative methods to reduce the ecological footprint of wood waste^20^. Solid-state reduction, as a one-step technique, represents a solvent-free synthesis and is compatible with green chemistry principles. Unlike liquid-phase routes, the solid-state technique confines aggregation through minimizing NPs mobility. This spatial restriction promotes uniform dispersion of NPs without the need for external stabilizers. Compared to other green synthesis approaches, the method offers improved simplicity, reproducibility, and integration of NPs within the support material^21^.

Among these advancements, selenium nanoparticles (SeNPs) have gained attention for their antioxidant and antimicrobial properties, significantly enhancing the durability of wood materials^22^. Studies have shown that treating wood with SeNPs improves antimicrobial resistance and even enhances color aesthetics^23^. The eco-friendly synthesis of SeNPs using plant-based or microbial methods further supports their sustainability, making them a promising solution for environmentally responsible wood treatments^24,25^. Additionally, epoxy resin has emerged as an effective alternative to traditional adhesives. Its exceptional properties and versatile applications have driven market growth. Epoxy-based panels comply with international standards, making them suitable for furniture applications^26,27^. By integrating green SeNPs and epoxy resin into pine wood sawdust (PS), the production of sustainable, eco-friendly particleboards becomes achievable, aligning with global sustainability efforts.

This study aims to fabricate a novel material based on pine sawdust (PS) deposited with SeNPs for the first time using a solid-state technique as a solventless and economic method without the need for stabilizers. Sodium hydrogen selenite as precursor for SeNPs and ascorbic acid as reductant were used in the in-situ synthesis of SeNPs on the surfaces of PS. This method enables the industrial sector to prepare a large amount of the treated sawdust with a high concentration of deposited SeNPs without the risk of aggregation compared to other traditional liquid methods, which require low quantities of NPs to avoid formation of large particles due to the high mobility and therefore, the potential aggregation. The antimicrobial activities were evaluated against the tested bacterial and fungal strains, which showed significant sensitivity to the SeNPs-PS samples. In addition, the color properties of the fabricated SeNPs-PS samples were determined to study the effect of the concentration of the deposited SeNPs on the color strength.

## Experimental sections

### Materials

Sawdust was collected from furniture-grade pine wood (*Pinus sylvestris*) manufactured in Damietta, Egypt; the wood itself is imported from CEDAR d.o.o. (Rijeka, Croatia). Sodium hydrogen selenite (NaHSeO₃; 96%) and ascorbic acid (C₆H₈O₆; 99%) were purchased from Sigma-Aldrich and used as received, without further purification. Agar-Agar powder (Agar GRG, WINLAB Laboratory Chemicals, UK) with purity ≥ 99%, and Nutrient Agar (NA) (Arena BioScien, Egypt) as a dehydrated culture medium with purity ≥ 99% were purchased for microbiological experiments.

### Procedures

#### Preparation ofsawdust

The sawdust was initially dried to a moisture content of less than 5%, following the procedures outlined in ASTM D4442-20, ISO 3130:1975^28,29^. It was then ground into fine particles using a mechanical grinder (Tencanm GJ-3, China) to achieve a particle size consistency of 80–100 mesh (~ 150–180 μm). The sawdust was subsequently divided into four groups, each treated with a different concentration of selenium nanoparticles (SeNPs).

#### Determination of PS moisture content

The moisture content (MC) of the PS was determined using the primary oven-drying method in accordance with ASTM D4442-20 and ISO 3130:1975^28,29^. Representative samples (3 ± 0.5 g) were weighed in triplicate using an analytical balance (A&D, HR-250 A, Tokyo, Japan), where the variation between successive measurements was maintained below 0.0001 to obtain the initial mass (*m*_1_​). The specimens were then placed in a forced-convection oven (Yamato, DKN-402 C, Tokyo, Japan) at a controlled temperature of 103 ± 2^∘^C.

The drying process was maintained until a constant mass was achieved, defined as a change in mass of less than 0.1% between two successive weightings at 2 h intervals. After drying, samples were transferred to a desiccator containing silica gel and allowed to cool to ambient temperature (25^∘^C ± 2^∘^C) to prevent hygroscopic moisture reabsorption. The final oven-dry mass (*m*_0_​) was recorded. The moisture content (*MC*) on an oven-dry basis was calculated using the following equation:

$$MC\left(\%\right)=\left(\frac{\left({m}_{1}-{m}_{0}\right)}{{m}_{0}}\right)\times100$$

Only PS batches achieving a moisture content of < 5% were utilized for the subsequent in-situ synthesis of SeNPs to ensure optimal precursor absorption and surface deposition uniformity.

#### Synthesis of SeNPs on the sawdust surface

**Fig. 1:**
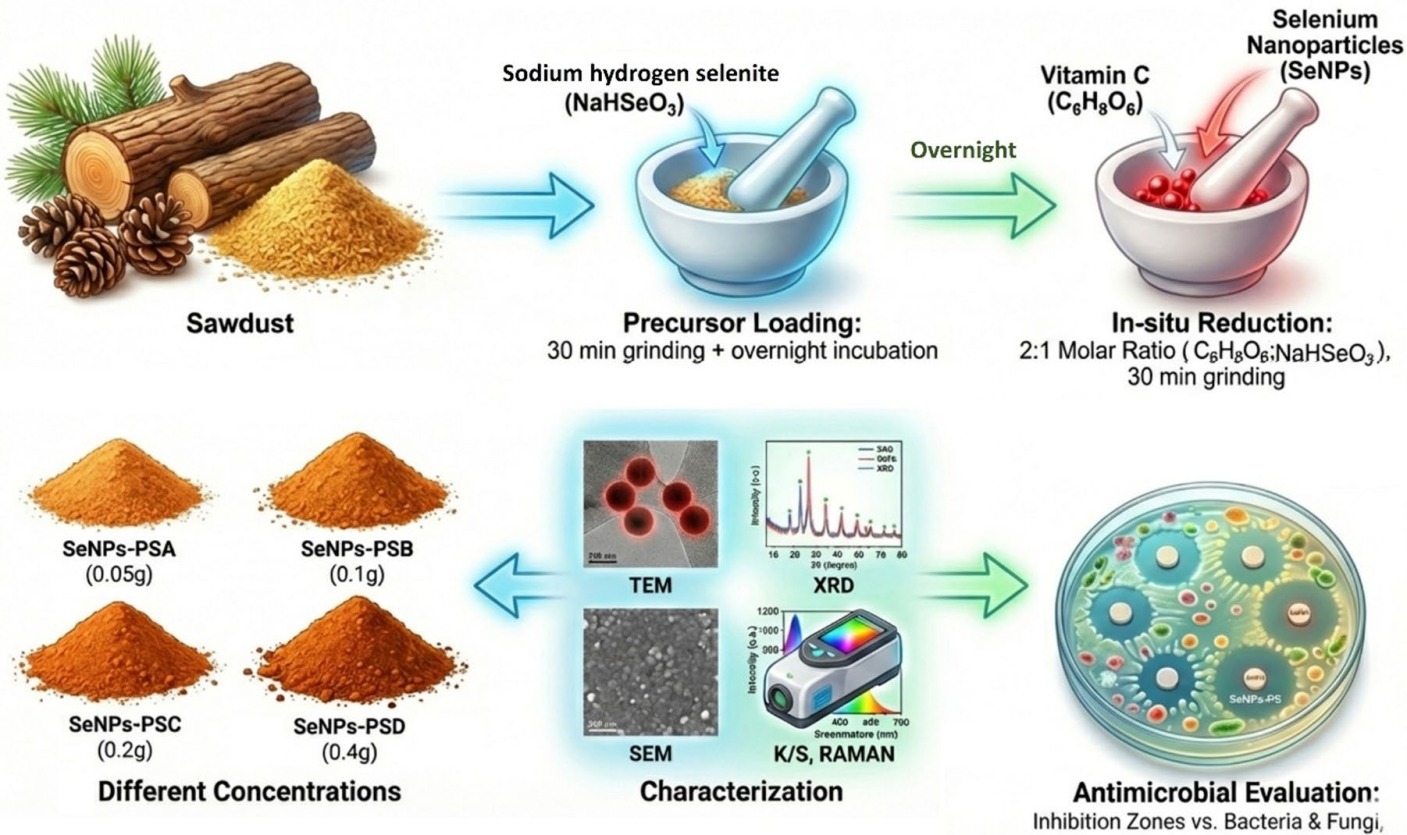
A schematic diagram for the solid-state synthesis of SeNPs deposited on the PS.

The SeNPs were synthesized directly on the surface of the sawdust through a one-step solid-state reduction technique at room temperature (25 °C ± 2), as a solvent-less method without stabilizers, as shown in Fig. 1. In this process, 2 g of powdered sawdust was mixed with different quantities (0.05, 0.1, 0.2, and 0.4 g) of sodium hydrogen selenite (NaHSeO_3_), as the precursor for SeNPs, and ground together for 30 min in a mortar. The mixture was then let overnight to encourage the deposition of selenium ions on the surface of the sawdust. In addition, ascorbic acid, as the reducing agent, was then added to this mixture at a molar ratio of 2:1 (ascorbic acid: NaHSeO_3_), as listed in Table 1, with further grinding for 30 min to allow the formation and direct deposition of SeNPs onto the sawdust surfaces at different concentrations to study the effect of the prepared NPs concentration on the size and, therefore, the antimicrobial and coloring properties.

**Table 1:** Sodium hydrogen selenite and ascorbic acid proportions required for 2 g of pine sawdust during solid-state synthesis of SeNPs in a one-step process on the sawdust surface.

NPs	NaHSeO₃ (g)	Ascorbic acid (g)
SeNPs-PSA	0.05	0.1167
SeNPs-PSB	0.1	0.2333
SeNPs-PSC	0.2	0.4667
SeNPs-PSD	0.4	0.9333

### Characterization of SeNPs and sawdust

#### TEM analysis

SeNPs were examined using a transmission electron microscope (JEOL, JEM 2100 F, Tokyo, Japan) at 200 kV to characterize their morphology and size. Samples of the SeNPs-PS powder were dispersed in ethanol, and the mixture was ultrasonicated for 10–15 min to break apart the particles and ensure a homogeneous suspension suitable for TEM imaging. A small drop of the dispersed sample was applied to a carbon-coated copper grid (400-mesh) and air-dried completely, as described in^30^. The dried grid was then ready for TEM characterization to analyze the morphology and size of SeNPs deposited on the sawdust surfaces.

#### X-ray diffraction (XRD)

An X-ray diffractometer (Bruker D8 ADVANCE, Karlsruhe, Germany) was used to determine the crystalline structure of the synthesized SeNPs deposited on the surfaces of PS.

#### Colorimetric analysis

The K/S values and color characteristics of SeNPs-PS specimens were measured using a spectrophotometer (Minolta CM-3600 A, Tokyo, Japan) and compared with those of the untreated sample used as a control.

#### Evaluation of antimicrobial activities of SeNPs-PS specimens

The antimicrobial activities of PS specimens and SeNPs-PS were evaluated against Gram-negative (G−ve) bacteria (*Pseudomonas aeruginosa* and *Escherichia coli*), Gram-positive (G + ve) bacteria (*Bacillus cereus* and *Micrococcus albus*), and fungi (*Aspergillus niger*, *Aspergillus flavus*, and *Aspergillus ochraceus*). The antimicrobial activities of PS were tested using the agar diffusion method. Wells of 8 mm diameter were created in nutrient agar plates using a cork borer. Bacterial suspensions were inoculated by streaking on nutrient agar plates, whereas fungal suspensions were inoculated on potato dextrose agar plates, following the recommendations of the Antibiogram Committee of the French Society of Microbiology (CASFM) and European Committee on Antimicrobial Susceptibility Testing (EUCAST). The wells were then filled with 30 mg of PS and SeNPs-PS, and the nutrient agar and potato dextrose agar plates were incubated at 37 °C for 24 h and 30 °C for four days, respectively.

The tests were performed in triplicate, and inhibition zones were examined after the incubation period. A positive result (+) was recorded if an inhibition zone of more than 0.5 mm was observed in all replicates; otherwise, the result was considered negative, as described by^31^. The experiment was conducted in the biotechnology laboratory of the Center for Excellence in Research of Advanced Agricultural Sciences (CERAAS), Damietta University, Egypt.

### Statistical analysis

The data were statistically analyzed using the Costat system for Windows, Version 6.311 (CoHort software, Monterey, CA 93940, USA) to get significant differences and standard deviation (SD). All multiple comparisons were first subjected to analysis of variance (ANOVA). Significant differences among treatment means were determined with Duncan’s new multiple range test at *p* = 0.05^32–34^.

## Results and discussion

### Characterization of synthesized Se‑NPs

### TEM analysis

**Fig. 2:**
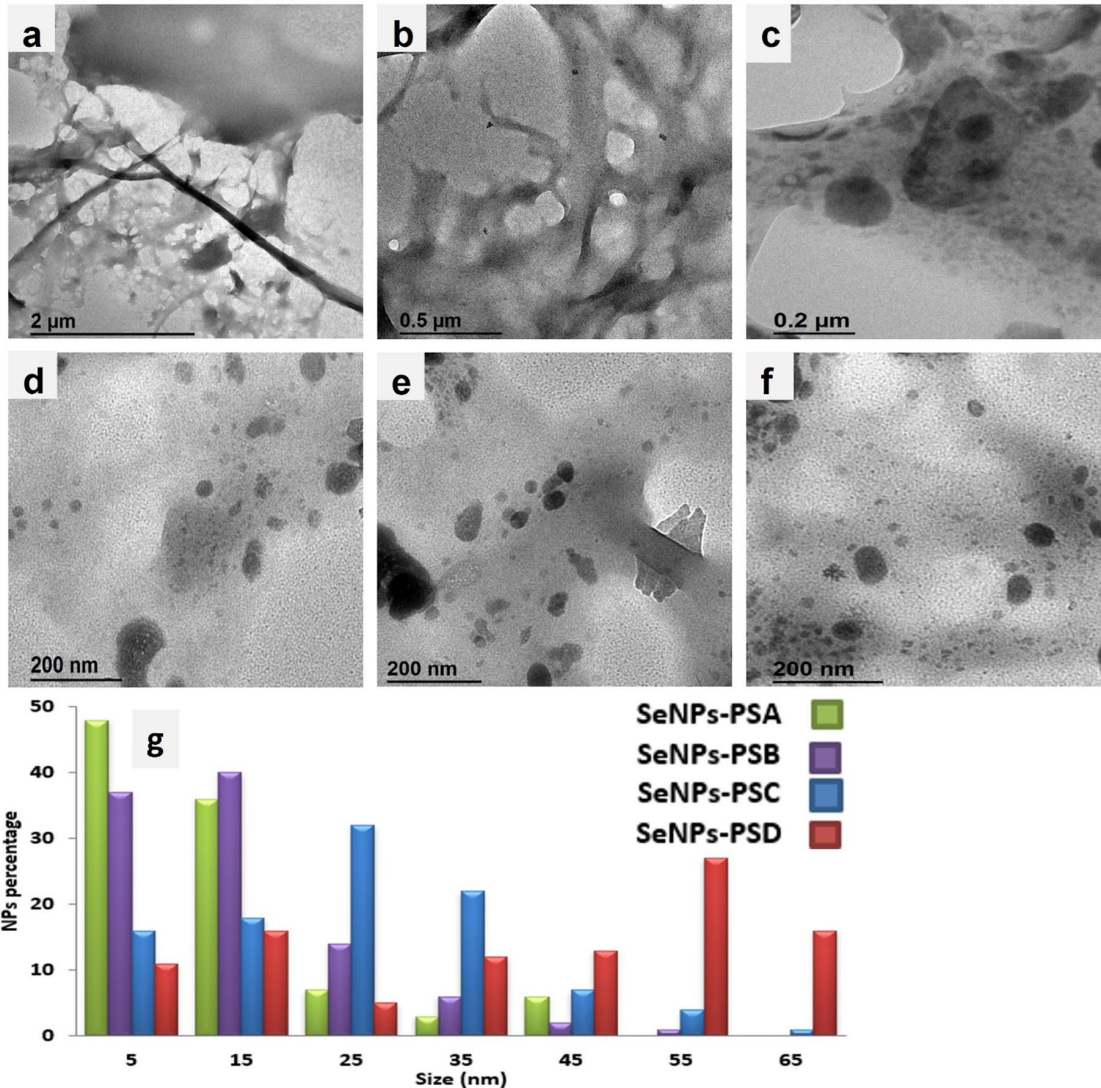
TEM micrographs of (**a**,** b**) blank PS, (**c**) SeNPs-PSA, (**d**) SeNPs-PSB, (**e**) SeNPs-PSC, and (**f**) SeNPs-PSD, in addition to (**g**) histogram of SeNPs sizes for the treated PS samples.

TEM micrographs of PS displayed surfaces free from deposited particles, as shown in Fig. 2(a, b). On the other hand, SeNPs-PS micrographs showed the deposition of SeNPs in spherical shape on the treated PS surfaces, as shown in Fig. 2(c–f). At lower concentrations (SeNPs-PSA and SeNPs-PSB), the deposited SeNPs were smaller and more sparsely distributed. Specifically, SeNPs-PSA, most NPs fall into smaller size ranges ~ 3–49 nm, as shown in Fig. 2(c). While SeNPs-PSB exhibited deposited SeNPs ranging from ~ 3–59 nm. The size of the deposited SeNPs was increased with the increase in SeNPs concentration, encouraging the aggregation of the particles deposited on the PS surfaces, as shown in the histogram in Fig. 2(g). In this respect, SeNPs-PSC and SeNPs-PSD showed larger SeNPs on the PS surfaces than other SeNPs-PS samples. SeNPs-PSD TEM micrographs showed a distribution dominated by larger NPs ~ 5–68 nm due to aggregation and the formation of larger particles, as displayed in Fig. 2(f).

Depending on the TEM images, a histogram of the SeNPs deposited on PS was obtained, as illustrated in Fig. 2(g). The width of the histogram bins was 10 nm and centered at 5, 15, 25, 35, 45, 55, and 65 nm. For example, all SeNPs with sizes ranging from 10 to 20 nm were expressed together as a bin of 15 nm. The size of the deposited SeNPs depended on the precursor (sodium hydrogen selenite) concentrations. SeNPs-PSA and SeNPs-PSB histograms showed that the most SeNPs deposited on the PS surfaces were around 5 and 15 nm, while SeNPs-PSC exhibited the highest SeNPs numbers around 25 and 35 nm. In addition, most of the SeNPs deposited on the SeNPs-PSD surfaces were expressed with bins of 55 and 65 nm, indicating that the increase in SeNPs concentration on the PS surfaces encouraged the aggregation, and therefore gradually showed an increase in SeNPs bins.

#### SEM and EDX analyses

**Fig. 3:**
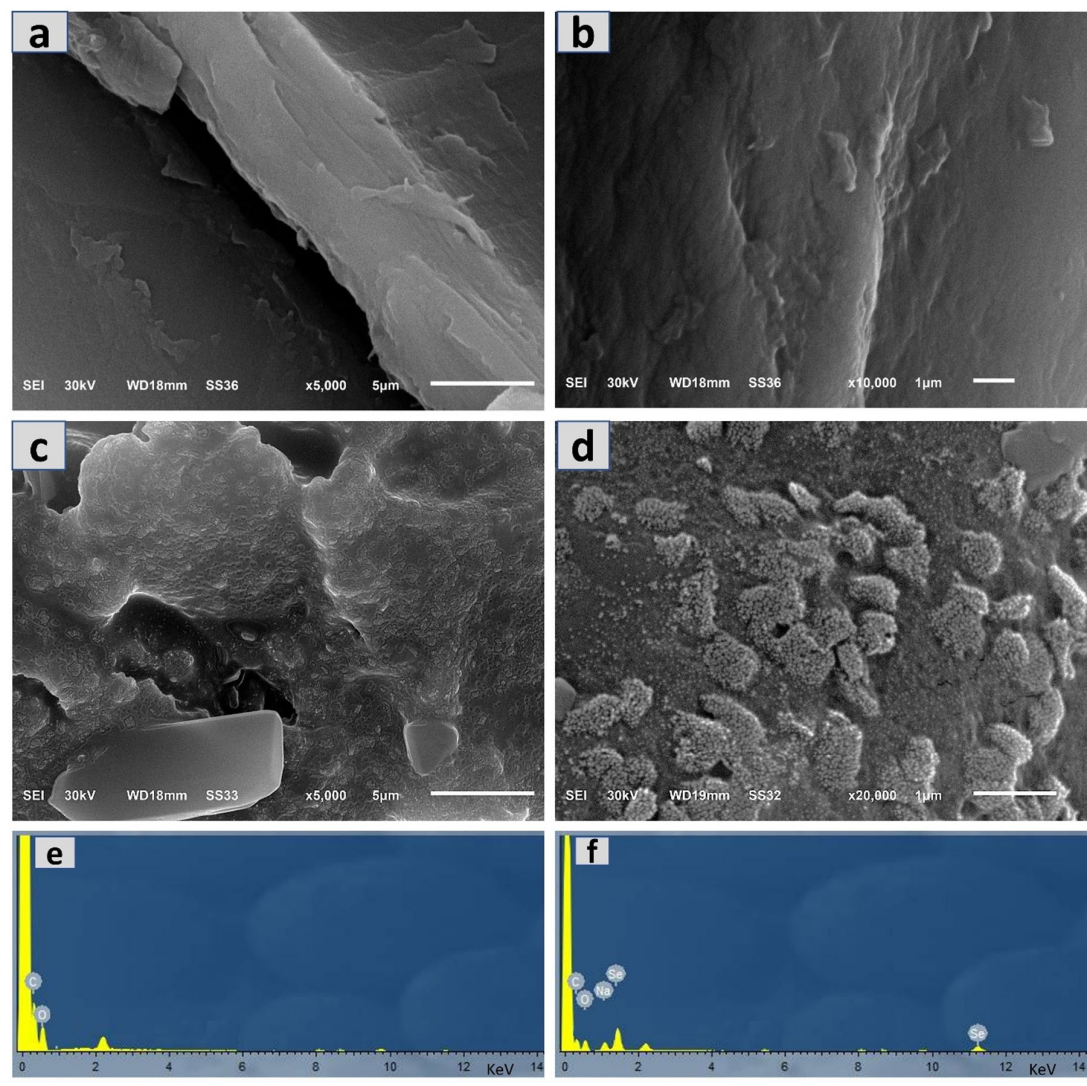
SEM micrographs of (**a**,** b**) blank PS, and (**c**,** d**) SeNPs-PSD, in addition to EDX analysis of (**e**) blank PS, and (**f**) SeNPs-PSD.

The surface morphology of PS and SeNPs-PS was examined using SEM analysis, as shown in Fig. 3(a–d). The SEM micrographs clearly demonstrate obvious morphological changes as a result of SeNPs deposition on the PS surface after solid-state synthesis. The obtained micrographs of PS at low and high magnifications (Fig. 3a and b) reveal the characteristic fibrous and layered architecture of lignocellulosic biomass. The surface appears relatively smooth with longitudinal grooves and compact fibrillar structures, corresponding to the aligned cellulose microfibrils included in the sawdust microstructure. No remarkable particles were observed at the nanoscale on the blank PS surface.

In contrast, SeNPs-PS micrographs after solid-state synthesis and SeNPs deposition exhibit pronounced morphological modifications (Fig. 3c and d). The surface becomes significantly roughened and partially covered by well-dispersed and spherical SeNPs, illustrating the sufficient deposition of SeNPs on the PS surface. In addition, the SEM micrographs ensured that the lignocellulosic matrix acts as a stabilizing and support material during SeNPs solid-state synthesis. The roughened surface and high surface coverage indicate strong interfacial interactions between SeNPs and the functional groups (e.g., –OH, –COOH) present in the PS surface^35^. These interactions enhance immobilization and avoid NPs migration from the surface, resulting in stable NPs deposition. Additionally, no evidence of severe structural collapse or degradation of the underlying fibrous framework is observed, confirming the applicability of one-step solid-state synthesis.

The EDX spectrum of the blank PS surface shows dominant peaks corresponding to carbon (C) and oxygen (O), as shown in Fig. 3(e). These elements are characteristic of lignocellulosic biomass, which is primarily composed of cellulose, hemicellulose, and lignin^36^. The strong C peak and moderate O peak confirm the organic nature of the substrate. No selenium-related signals are observed in this spectrum, indicating the absence of SeNPs on the surface of blank SP. On the other hand, the EDX spectrum of the SeNPs-PS clearly shows a distinct selenium (Se) peak, typically observed around ~ 1.37 keV (Se Lα) and ~ 11.2 keV (Se Kα), as shown in Fig. 3(f). The presence of this peak confirms the successful formation and deposition of SeNPs onto the SeNPs-PS surface. The continued presence of C and O peaks indicates that the lignocellulosic matrix remains intact after the synthesis process. Additionally, sodium (Na) was detected, which can be attributed to residual sodium from the sodium hydrogen selenite precursor used in the solid-state synthesis.

#### Raman analysis

**Fig. 4:**
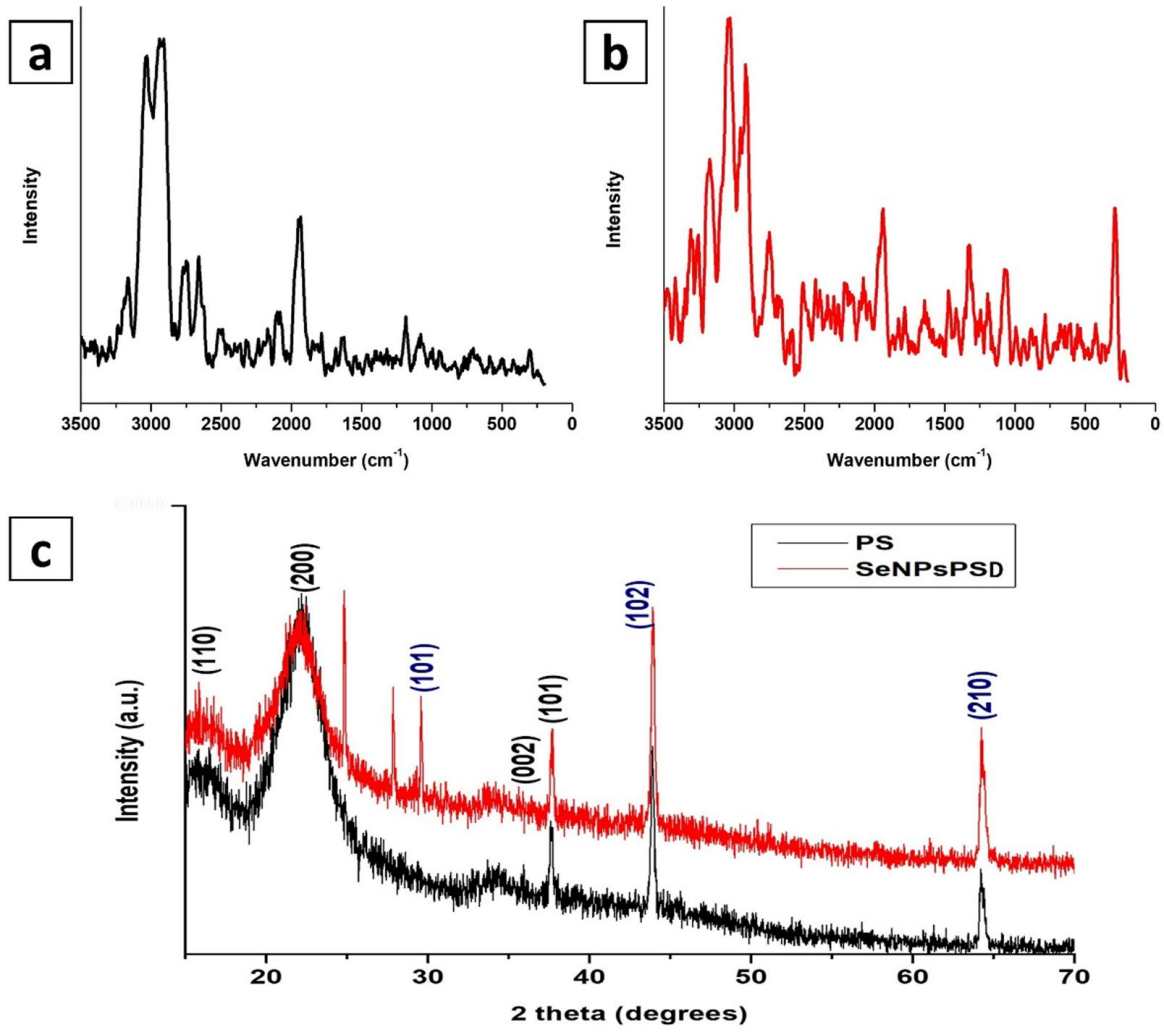
Raman spectra of (**a**) PS, and (**b**) SeNPs-PSD, and (**c**) XRD patterns of blank PS and SeNPs-PSD.

PS is mainly composed of cellulose, hemicellulose, and lignin, and its Raman spectrum reflects these components, as shown in Fig. 4(a). Two strong bands observed at 2920 and 3030 cm^− 1^ correspond to C–H stretching vibrations from cellulose and hemicellulose. In addition, peaks at 1645 and 1475 cm^− 1^ are found, which may be due to C = C stretching from lignin and CH₂ bending (cellulose/lignin), respectively. Additional bands present at 1325 and the region from 1095 to 1120 cm^− 1^ indicated the presence of C–H bending in cellulose, and C–O–C and C–O stretching of cellulose backbone, respectively. Furthermore, skeletal vibrations of polysaccharides are detected as a result of small peaks in the region below 1000 cm^− 1^. In general, these peaks confirm the typical lignocellulosic structure of pine sawdust^37^. In the case of SeNPs-PS, a sharp peak was observed in the region from 250 to 300 cm^− 1^, confirming the formation of SeNPs on the surface of the PS and the sufficient deposition of the NPs. The intensity of peaks at 2920 and 3030 cm^− 1^ decreased may be owing to the deposition of NPs on the surface, as shown in Fig. 4(c)^38^.

#### XRD analysis

The XRD patterns revealed remarkable differences between the blank PS and the SeNPs-PS samples, as shown in Fig. 4. The blank sample showed peaks at 15.84, 22.18, 35.94, 37.6, 43.92, and 64.2º, which may correspond to the crystal planes of (110), (200), (002), (101), (102), and (210), respectively^39^. The Blank PS exhibited a broad peak around 20–30° 2θ, indicating an amorphous structure. The crystallinity of pine is determined by its composition, as cellulose is the only crystalline component, whereas both hemicellulose and lignin are amorphous^40^. In addition, the XRD pattern of the fabricated SeNPs-PS showed the same peaks of PS and additional peaks regarding the SeNPs deposited on the surface and the products of the chemical reaction in the solid-state technique. In this respect, SeNPs-PS showed peaks at 43.92 and 64.2º with higher intensity compared to those of PS due to the formation of SeNPs, as displayed in Fig. 4. Additionally, an obvious additional peak was observed for SeNPs at 29.34º, corresponding to a crystal plane of (101). This is in good agreement with the data documented in the JCPDS standard card (No. 06–0362) for SeNPs, confirming the deposition of SeNPs on the surface of PS^41^. The peaks observed at 27.86 and 24.48º may be due to the presence of ascorbate salt as a product of the reaction^42^.

#### Colorimetric Analysis

**Fig. 5:**
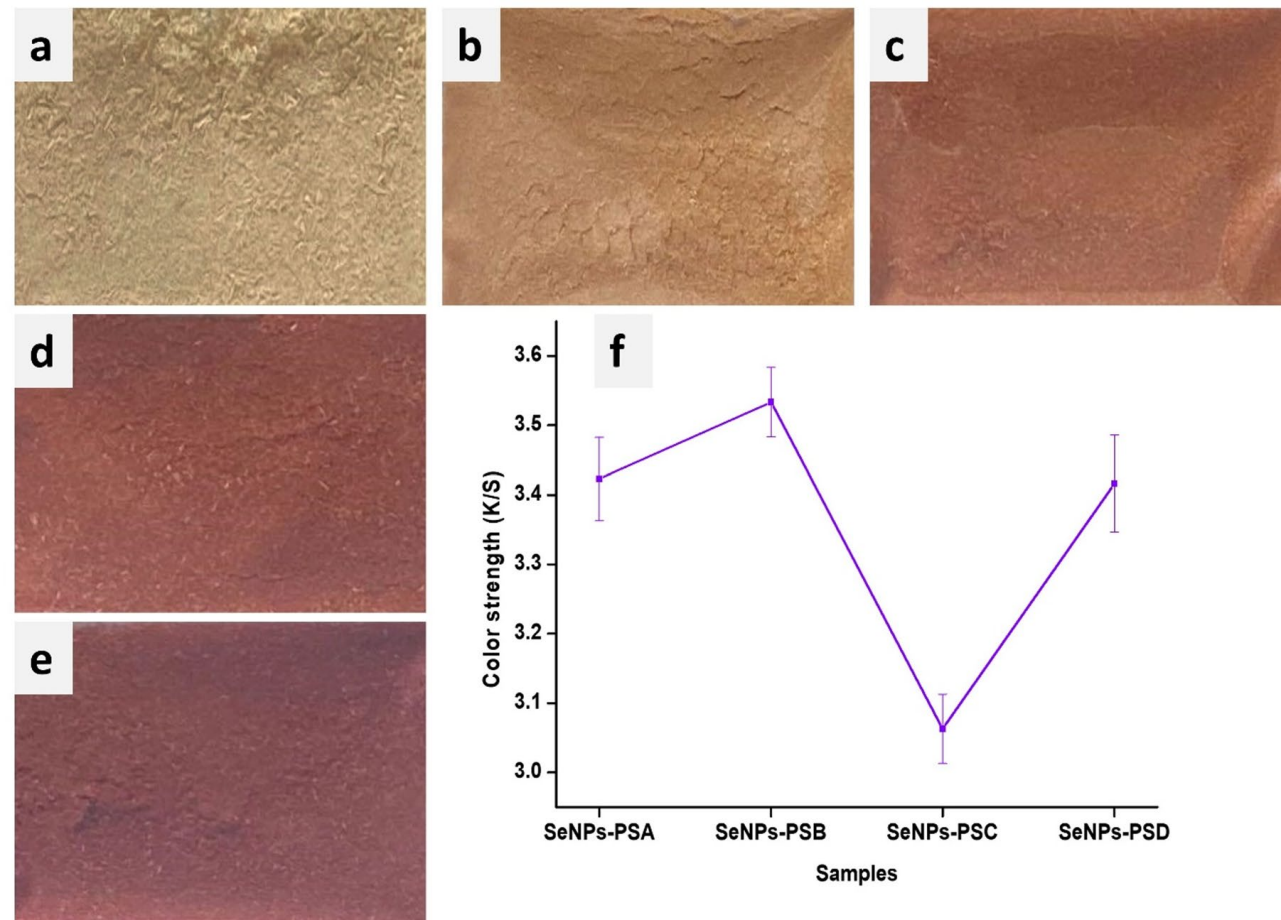
Images of PS surfaces of (**a**) blank PS, (**b**) SeNPs-PSA, (**c**) SeNPs-PSB, (**d**) SeNPs-PSC, (**e**) SeNPs-PSD, and (**f**) color strength of the fabricated SeNPs-Ps samples.

The colorimetric properties of SeNPs-PS samples were analyzed spectrophotometrically to evaluate the effect of SeNPs on the surface color of PS. Upon treatment with SeNPs, the PS specimens exhibited a range of orange hues, from light to medium and dark orange, depending on the SeNP concentration applied, as illustrated in Fig. 5. This indicated that SeNPs were evenly deposited on the surfaces of the treated PS samples, exhibiting good homogeneity. In contrast, the blank PS samples remained clear and free of NPs. The analysis revealed that the color intensity of the PS specimens increased proportionally with the increase in SeNPs concentration, demonstrating a direct relationship between NPs deposition and color enhancement.

The color strength (K/S) of PS was measured after treatment with SeNPs at different concentrations, as shown in Fig. 5(f). At a low SeNPs concentration (SeNPs-PSA), the treated PS samples exhibited relatively low K/S values. As the SeNPs concentration increased, the K/S value rose, with the samples developing a more pronounced reddish tone. The highest K/S value was observed for SeNPs-PSB, which was significantly greater than SeNPs-PSA. However, at higher concentrations (SeNPs-PSC and SeNPs-PSD), the K/S values decreased. This suggests that additional SeNPs amount than that of SeNPs-PSB do not further enhance the color strength and may even cause NPs aggregation, reducing their K/S values. Previous study revealed similar results and confirmed that as the SeNPs concentration increases, the interparticle collision frequency increases, promoting agglomeration and the subsequent formation of larger particle clusters, leading to reduced K/S due to crowding and reduced active surface area^43^.

The K/S of SeNPs-PS at varying concentrations was measured. The K/S value began at approximately 3.42 for SeNPs-PSA and increased to a peak of 3.53 in the case of SeNPs-PSB, indicating the highest color strength at this concentration. Following this, the K/S value of SeNPs-PSC decreased slightly to 3.39, reflecting a drop in color strength. Beyond this concentration, the SeNPs-PSD K/S value stabilized around 3.42, as shown in Fig. 5(f). This plateau effect may be attributed to the aggregation of NPs at higher concentrations, which reduces their effectiveness, as evident in Fig. 5. This saturation point occurred because the PS capacity to absorb NPs became limited. Further additions may lead to aggregation, as confirmed by TEM analysis, which did not contribute to increased color strength and can even reduce it^24^.

NPs, due to plasmon resonance, exhibit distinct colors. For example, ZnONPs, which are optically white and primarily functional, while AgNPs may induce undesirable darkening of wood substrates. However, SeNPs exhibit tunable orange-red coloration (K/S up to 3.53), which is desirable for furniture and wood applications. From an environmental perspective, SeNPs are generally reported to exhibit lower cytotoxicity and reduced ion-leaching concerns compared with AgNPs systems, suggesting a potentially safer profile for decorative-functional wood applications when used within controlled concentration ranges^44^.

#### Evaluation of the antibacterial activities

**Fig. 6:**
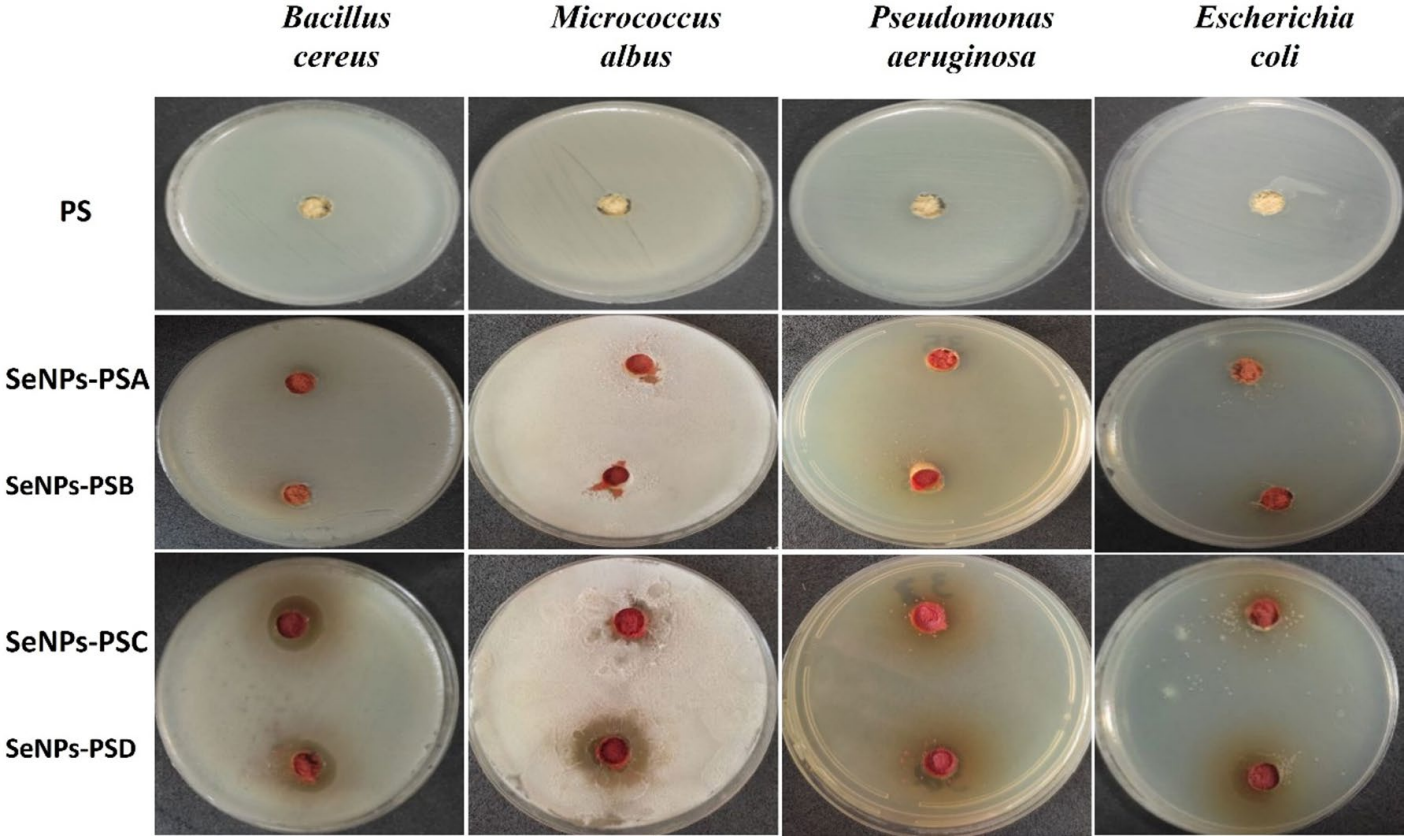
Evaluation of the antibacterial properties of PS and SeNPs-PS samples against the tested bacterial strains.

**Fig. 7:**
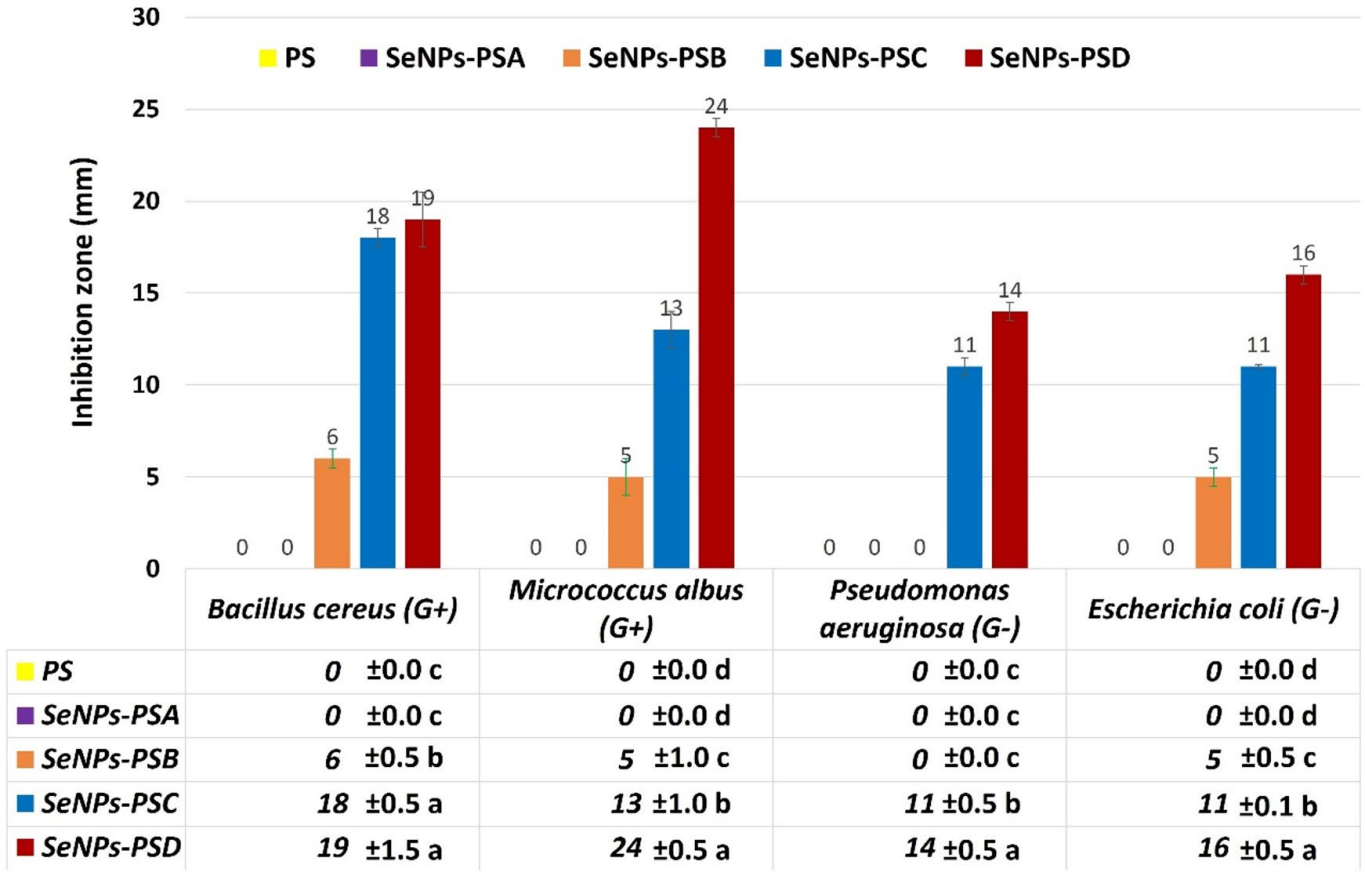
Histogram of the antibacterial activities of PS and SeNPs-PS samples.

The antibacterial activities of PS treated with SeNPs at different concentrations were evaluated against various bacterial strains, including *Escherichia coli* and *Pseudomonas aeruginosa* as gram-negative (G-ve) bacteria and *Bacillus cereus* and *Micrococcus albus* as gram-positive bacteria (G + ve). The results shown in Figs. 6 and 7 illustrated that the SeNPs-PS exhibited promising antibacterial activities against the tested bacterial strains compared to blank PS. In addition, SeNPs-PSD was more effective than other SeNPs-PS samples against *Pseudomonas aeruginosa*,* Escherichia coli*, *Bacillus cereus*, and *Micrococcus albus*, showing inhibition zones of 14, 16, 19, and 24 mm, respectively. The antibacterial activities of the SeNPs-PS samples increased with the increase in the concentration of SeNPs deposited on the PS surfaces. In this respect, SeNPs-PSA did not exhibit antibacterial activities against the tested bacterial strains, while SeNPs-PSB showed remarkable antimicrobial activities against the tested bacterial strains, with inhibition zones ranging from 5 to 6 mm, except against *Pseudomonas aeruginosa*, which was the most resistant bacterial strain against the SeNPs-PS samples and showed complete resistance against SeNPs-PSB without any observed inhibition zone, as displayed in Fig. 6. In addition, SeNPs-PSC showed a significant increase in the determined inhibition zones compared to SeNPs-PSA and SeNPs-PSB.

Additionally, a comparison was performed with another study^38^ conducted under identical experimental conditions (agar diffusion method, same three bacterial strains of *Bacillus cereus*,* Escherichia coli*,* Pseudomonas aeruginosa*, and incubation parameters), in which standard antibiotics (tetracycline and ciprofloxacin) were evaluated. The inhibition zones recorded for the SeNPs-PS (5–24 mm) were found to be similar to or higher than those obtained for tetracycline (15–19 mm) and ciprofloxacin (17–21 mm) against the same bacterial strains. In some cases, particularly against *Bacillus cereus* and *Pseudomonas aeruginosa*, the SeNPs-PS inhibition values were within or exceeded the range of the common standard antibiotics. These findings highlight the strong antibacterial performance of the functionalized sustainable composite and confirm its potential as an effective alternative antimicrobial material.

In addition, untreated PS showed no antibacterial activity against any of the tested bacterial strains, as shown in Fig. 6. Similar trends have been reported, where gram-negative bacteria have an outer membrane that antimicrobials must pass through to attack the bacteria^31^. Because of the lack of this outer layer in G + ve bacteria, more of the tested samples could provide resistance against G + ve bacteria than G-ve bacteria^45^. The high reactivity of SeNPs, owing to their large surface-to-volume ratio, results in intrinsic targeted antimicrobial efficiency, even when they are applied in small amounts^46^.

#### Evaluation of the antifungal activities

**Fig. 8:**
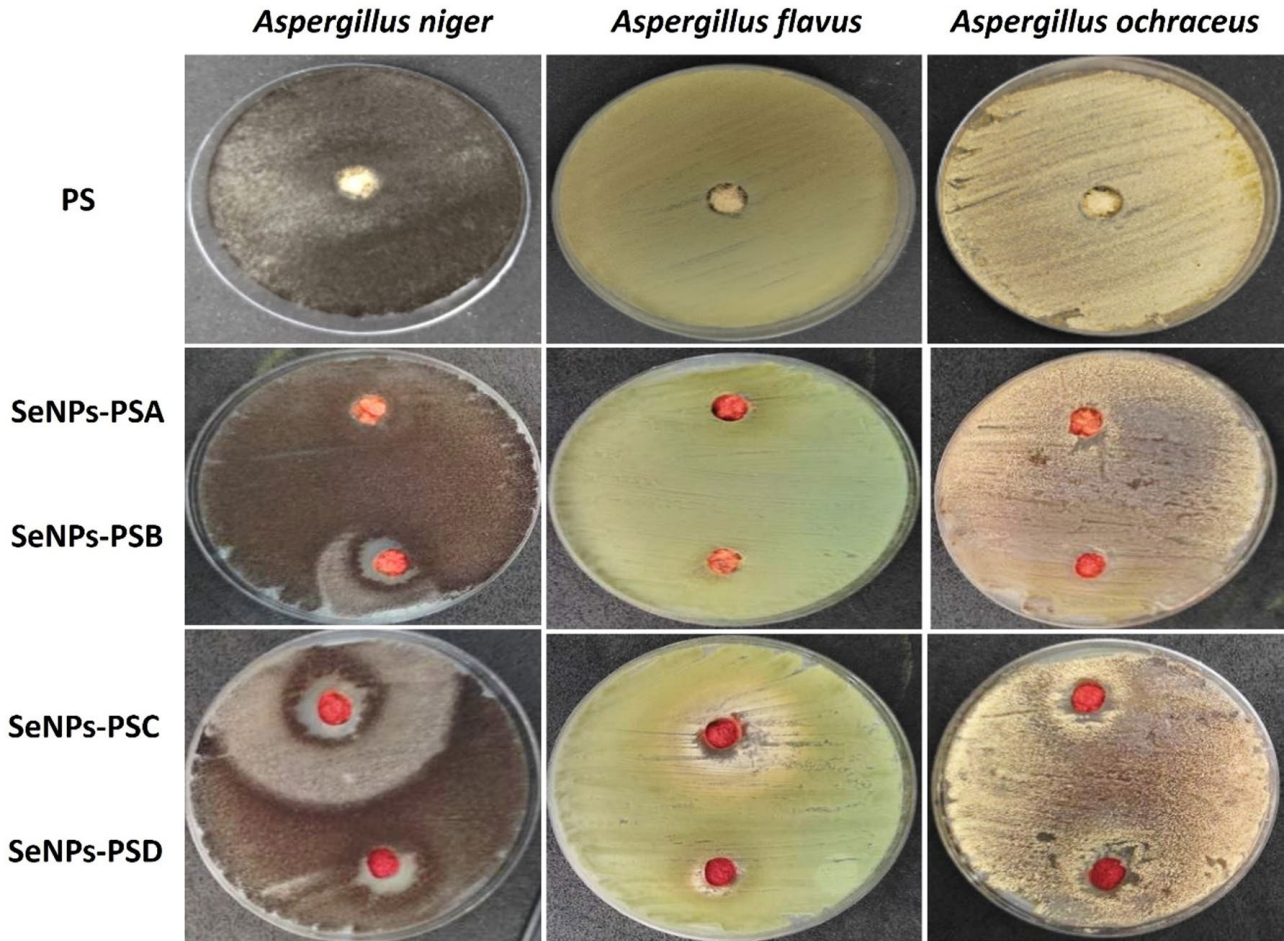
Evaluation of the antifungal properties of PS and SeNPs-PS samples against the tested fungal strains.

**Fig. 9:**
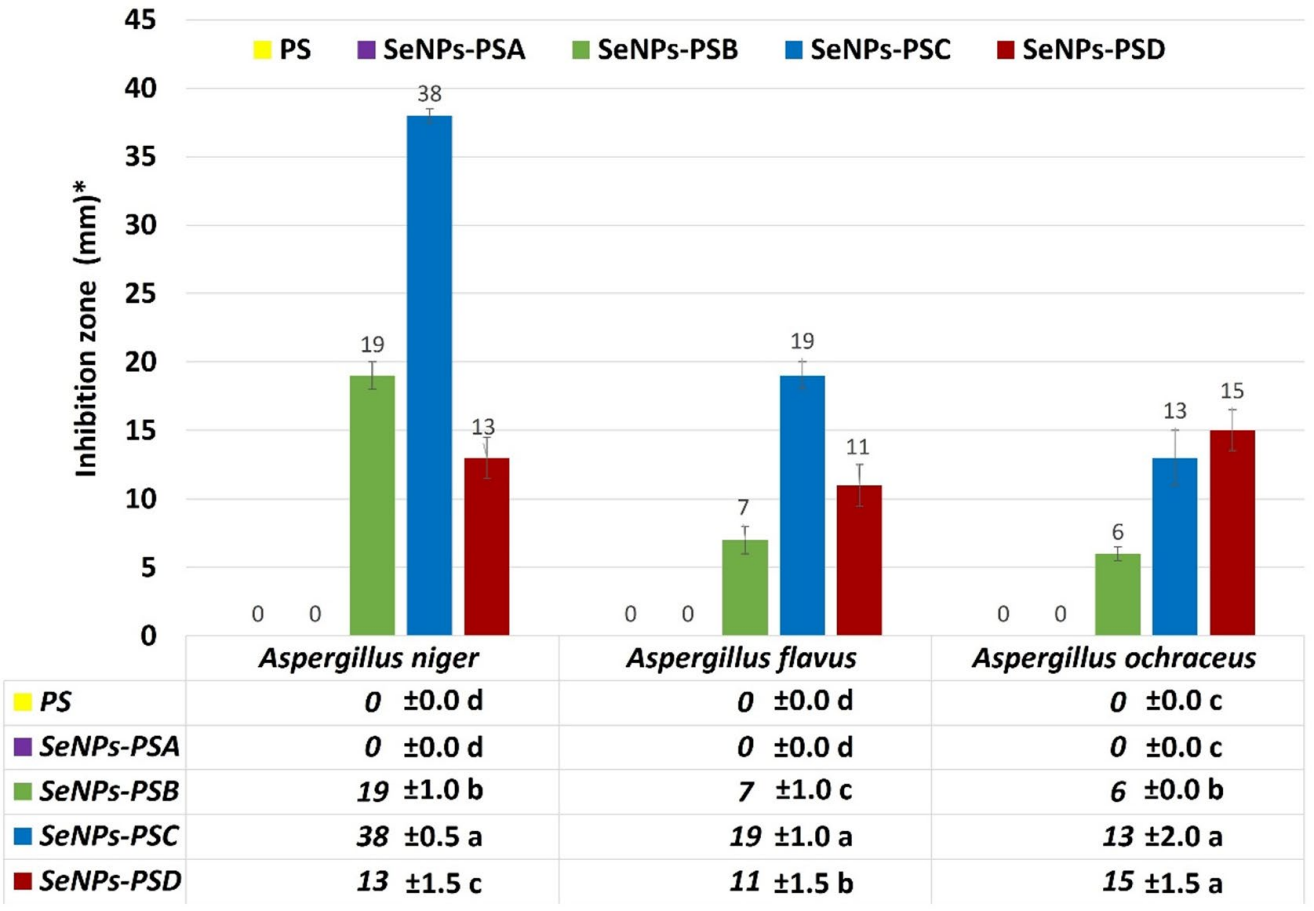
Histogram of the antifungal activities of PS and SeNPs-PS samples.

The antifungal activity of PS treated with SeNPs was evaluated against *Aspergillus niger*, *A. flavus*, and *A. ochraceus*. The obtained data (Figs. 8 and 9) clearly demonstrated a concentration-dependent inhibition pattern, where increasing SeNP concentrations led to progressively larger inhibition zones. No inhibition was observed in the control (PS), indicating that the antifungal activity was solely due to the SeNPs. At the lowest concentration (SeNPs-PSA), no detectable inhibition occurred for any fungus, whereas increasing the concentration in SeNPs-PSB resulted in noticeable inhibition, most pronounced against *A. niger* (19 ± 1.0 mm). The most significant antifungal effect was observed with SeNPs-PSC (38 ± 0.5 mm for *A. niger*, 19 ± 1.0 mm for *A. flavus*, and 13 ± 2.0 mm for *A. ochraceus*), as shown in Fig. 8. Interestingly, a slight reduction in the inhibition zones of *A. niger* (13 ± 1.5 mm) was observed with SeNPs-PSD, as shown in Fig. 9, suggesting possible nanoparticle aggregation or decreased bioavailability at very high concentrations, a phenomenon previously noted by^47,48^. Among the tested fungi, *A. niger* was the most sensitive to SeNPs, followed by *A. flavus* and *A. ochraceus*. This variability may result from differences in the cell wall composition, hydrophobicity, and melanin content. The greater tolerance of *A. ochraceus* could be attributed to its thicker or more melanized cell walls, which reduce nanoparticle penetration. Similar species-dependent susceptibility patterns have been reported by^49,50^. In this study, the SeNPs-PSC showed a remarkable inhibition zone (38 mm) against *A. niger.* This performance significantly exceeds the standard values reported for common antifungal agents such as fluconazole and nystatin, tested by^51^, which showed inhibition zones between 15 and 22 mm against *Aspergillus* species in diffusion assays. This highlights the superior potential of the modified sawdust as a potent antifungal surface.

The antifungal activity of SeNPs-PS samples is attributed to a dual mechanism: electrostatic adhesion to fungal cell walls, which compromises membrane integrity, and the subsequent generation of reactive oxygen species (ROS). These ROS induce oxidative stress and lipid peroxidation, leading to cellular dysfunction and death^52^. This mechanism supports the optimal efficacy observed in SeNPs-PSC, representing a critical balance between sufficient ROS induction and effective NPs dispersion, effectively avoiding the agglomeration-related efficacy loss seen at higher concentrations. The inhibition zones obtained in this study (6–38 mm) are in good agreement with previous findings.^50^ reported inhibition zones around 15 mm for *A. niger* using 200 µg/mL SeNPs, while^47^ found 12–18 mm against *Aspergillus* spp., depending on the nanoparticle source and concentration. The results of the present study, therefore, confirm the potent antifungal potential of SeNPs, particularly at optimized concentrations. In this study, the rough SeNPs-coated surface, as shown in Fig. 3(c and d), increases interaction with fungal hyphae. SeNPs attach to fungal cell walls, leading to membrane distortion, structural collapse, and leakage of intracellular contents^49,50^.

The antifungal assay plates of *Aspergillus niger* treated with SeNPs-PSB and SeNPs-PSC revealed a characteristic three-zone inhibition pattern consisting of a clear central zone, a regrowth area, and an outer inhibition halo. This pattern is consistent with the Eagle effect, also known as a paradoxical growth phenomenon. Diffusion gradient SeNPs diffuse through the agar, forming different concentration zones. Variable fungal response: At high SeNPs concentrations, fungal spores are killed; at moderate levels, growth may be delayed or weakened; at lower levels, inhibition appears again, possibly due to delayed nanoparticle toxicity or ionic effects, in which increasing the antimicrobial concentration beyond an optimal point results in reduced inhibition or partial fungal regrowth^53,54^. As the distance from the application site increased, the SeNP concentration progressively decreased, generating spatially distinct exposure zones. At very high concentrations near the well, complete fungal inhibition occurred. At intermediate, sublethal concentrations, partial regrowth was observed, likely due to adaptive stress responses and insufficient nanoparticle penetration. At lower concentrations, dispersed NPs may penetrate more effectively and exert delayed toxicity, resulting in growth suppression again. This concentration-dependent response explains the formation of the three-zone antifungal pattern and is consistent with the Eagle effect and hormetic-like behavior reported for metal-based nanoparticles^52^. Furthermore, the diffusion gradient of SeNPs across the agar medium may have created variable exposure zones, as shown in Fig. 8. Intermediate concentrations are often more effective due to balanced nanoparticle dispersion and their interactions with the fungal membrane, whereas excessive concentrations near the application site may lead to nanoparticle clustering and reduced penetration^55^. Similar paradoxical antifungal patterns have been reported for *Candida* and *Aspergillus* species treated with metal-based nanoparticles, confirming that nanoparticle size, charge, and concentration critically determine their biological efficacy^50^.

Several types of NPs, such as AgNPs and ZnONPs, were widely reported as additives in wood panels and coatings due to their antimicrobial properties. Previous studies have shown that ZnONPs powder exhibited antibacterial activity^56^, reaching a maximum inhibition value of approximately 10.5 mm against *E. coli*, although this can vary slightly depending on the NPs loading and the type of microorganism. In addition, AgNPs were used for bacterial decontamination^57^. At concentrations of 0.5–8.5%, they showed effectiveness against *E. coli* and *S. aureus*. Notably, Ag-doped ZnONPs at various concentrations displayed outstanding antibacterial activity, outperforming the other tested samples, with inhibition zones ranging from 8.7 to 12 mm against *E. coli* and 9.7–12.5 mm against *B. cereus*^58,59^. However, solid state approach was not used before for the treatment of PS using SeNPs. SeNPs-PSC system demonstrated pronounced, concentration-dependent antifungal activity, reaching 38 mm against *A. niger* and 19 mm against *(A) flavus*, exceeding many reported ZnO-based systems and approaching AgNPs performance levels. For antibacterial activity, SeNPs-PSD achieved inhibition zones of 14, 16, 19, and 24 mm against *P. aeruginosa*, *E. coli*, *(B) cereus*, and *M. albus*, respectively, indicating strong broad-spectrum activity against both Gram-positive and Gram-negative strains.

## Conclusion

This study confirmed the hypothesis that a solvent-free solid-state reduction method can enable controlled in-situ synthesis and deposition of selenium nanoparticles (SeNPs) onto pine sawdust (PS) without stabilizers while minimizing aggregation. The process effectively transformed waste sawdust into a bioactive, value-added material with both aesthetic and antimicrobial functionalities. TEM, XRD, EDX, and Raman analyses confirmed the successful formation of crystalline SeNPs (3–68 nm) with tunable size distributions depending on precursor concentration. The incorporation of SeNPs imparted outstanding coloration to the sawdust, with maximum color strength observed for SeNPs-PSB, beyond which relative aggregation reduced optical performance. The SeNPs-PS composites exhibited strong antimicrobial efficacy against a broad range of microorganisms, including Gram-positive and Gram-negative bacteria, as well as fungal strains. Notably, *A. niger* showed the highest susceptibility, with inhibition zones reaching up to 38 mm for SeNPs-PSC. The main innovation lies in introducing a scalable, environmentally friendly, and low-cost approach for valorizing wood waste into high-value functional biomaterials with dual antimicrobial and coloration properties. This green strategy opens new possibilities for integrating SeNPs-modified sawdust into sustainable wood composites, active packaging, and antimicrobial surface coatings, contributing to circular bioeconomy and eco-innovative material design. In addition, this study enables the fabrication of large quantities of sawdust deposited with a high concentration of SeNPs without aggregation, which enhances its applicability in the industrial sector.

## Data Availability

All data supporting the findings of this study are available within the paper.
